# Maternal And Paternal Differences in Parental Stress and Children’s Autistic Features Among Parents of Preschool Autistic Children

**DOI:** 10.1177/13623613261427131

**Published:** 2026-03-26

**Authors:** James Rufus John, Anna Chua, Valsamma Eapen

**Affiliations:** 1 University of New South Wales, Australia; 2 Ingham Institute for Applied Medical Research, Australia; 3 South Western Sydney Local Health District, Australia

**Keywords:** autism, CALD, child behaviour, gender differences, parental stress, quality of life

## Abstract

Parents of autistic children experience varying levels of stress, with only few studies examining gender-specific differences and their determinants. This study examined the sociodemographic and clinical factors associated with stress among mothers and fathers of preschool-aged autistic children in Australia. Data were analysed from 516 parents of preschool-aged autistic children enrolled in six Autism Specific Early Learning and Care Centres. Multilevel regression models assessed associations between maternal and paternal stress and sociodemographic factors, parental quality of life, and child’s clinical characteristics, while adjusting for key covariates, and accounting for clustering of parent responses within children. Among the sample, 465 mothers and 216 fathers completed the Parental Stress Index–Fourth Edition–Short Form questionnaire, representing 516 unique children, with some children having responses from both parents. Findings showed that culturally and linguistically diverse status was protective against stress for both parents, but higher level of education was protective only against maternal stress. Maternal stress was influenced by a broad range of child’s autistic traits, including behavioural differences, social communication, repetitive behaviours and adaptive functioning, whereas paternal stress was only associated with behavioural differences. These findings highlight gender-specific factors affecting stress and reinforce the need for tailored supports for families of autistic preschool children.

**Lay Abstract:**

Many parents of autistic children experience high levels of stress. While mothers and fathers may face these challenges differently, only a few studies have explored gender-specific differences in parental stress. This study looked at the factors linked to stress in both mothers and fathers of preschool-aged autistic children in Australia. We analysed data from 516 parents whose children were enrolled in six Autism Specific Early Learning and Care Centres nationwide in Australia. Mothers reported higher overall stress than fathers. Parents from a culturally and linguistically diverse background or those who had higher education levels tended to report lower stress. On the other hand, greater child behavioural difficulties, challenges in social communication and poorer parental quality of life were linked to higher stress levels. These findings highlight the need for tailored, culturally sensitive supports for families, especially during the early years when children are starting intervention and parents are adapting to new caregiving demands.

## Introduction

Autism spectrum disorder (ASD) (hereafter referred as autism) is a neurodevelopmental condition characterised by persistent differences in social communication and the presence of restricted repetitive behaviours (American Psychiatric Association., 2022). It is estimated to impact approximately 28.3 million individuals worldwide with prevalence rates ranging from 1% to as high as 3.2% (Barbaro et al., 2022; Li et al., 2022; Shaw, 2025; Talantseva et al., 2023; Zeidan et al., 2022). Autistic children present with diverse profiles and varying levels of health and developmental needs, which may differ according to developmental stage, cognitive functioning, co-occurring conditions, and the availability of support (Al-Beltagi, 2021; May et al., 2020; Mosner et al., 2019). Beyond health considerations, autistic children are more likely to experience barriers in educational environments, including impacts on learning experiences, social participation, exposure to bullying, and difficulties in school attendance (Keen et al., 2016; Lassen et al., 2022; Munkhaugen et al., 2019; Park et al., 2020). These health and educational challenges can interact to adversely impact overall life outcomes, both in the short and long term (Chen et al., 2015; Steinhausen et al., 2016). Therefore, early identification and access to appropriate supports are crucial in ensuring optimal developmental outcomes.

Caring for an autistic child is associated with significant and ongoing parental wellbeing issues, with evidence showing a bidirectional relationship between autistic features and parental stress (Rodriguez et al., 2019; Zaidman-Zait et al., 2014). Several studies have shown that parents of autistic children report significantly higher stress than parents with non-autistic children and children with other disabilities, and consequently worse mental health, including anxiety and depression (Alibekova et al., 2022; Eapen & Guan, 2016; Enea & Rusu, 2020; Lievore et al., 2024). Furthermore, evidence indicates that autism is associated with notable economic impacts, with annual healthcare costs for autistic children reported to be four times higher than for non-autistic children (US$14,061 vs 3020) (Lavelle et al., 2014). In addition, indirect economic effects can occur when caregiving responsibilities influence parents’ or carers’ work participation, with an estimated annual impact of AU$34,900 in Australia (Horlin et al., 2014). Moreover, parents may face reduced employment opportunities, absenteeism, and low productivity due to a complex array of factors related to caring for an autistic child (Cidav et al., 2012; Liao & Li, 2020; Lynch et al., 2023). These intersecting health, social, and economic pressures highlight the importance of understanding the factors that contribute to parental stress.

Differences in child’s autistic features such as internalising behaviours (e.g. anxiety, emotional withdrawal, somatic complaints), externalising behaviours (e.g. aggression, hyperactivity, impulsivity, and oppositional behaviours), restricted and repetitive behaviours, and cognitive functioning can influence parental stress and wellbeing (Clauser et al., 2021; Rodriguez et al., 2019). Evidence suggests that internalising behaviours are often less overt but may contribute to cumulative effects of parental stress and wellbeing through ongoing concern and monitoring of needs (Rose et al., 2018). On the other hand, externalising behaviours are more disruptive to daily functioning and have been consistently linked to higher caregiving demands and parental distress (Morgan et al., 2005). Further, restricted and repetitive behaviours impact parental stress by limiting flexibility and increasing challenges around routines and transitions (Harrop et al., 2016), while cognitive difficulties can increase parental stress by requiring more intensive day-to-day support, reducing children’s independence, and thereby heightening parents’ concerns about learning, future functioning, and long-term care needs (Kennedy, 2012). Although previous studies have linked children’s behavioural and cognitive difficulties to parental stress, less is known about how specific behavioural domains differentially affect mothers’ and fathers’ stress and wellbeing. Understanding these parent- and domain-specific associations is essential for informing more targeted, equitable, and family-sensitive interventions.

Sociodemographic and familial factors, including cultural and linguistic diversity (CALD) status (also referred to as those from non-English speaking background) and socioeconomic status, may also shape parental experiences of stress and access to supports due to limited social acceptance and autism-related stigma (Kinnear et al., 2016; Papadopoulos, 2021). Despite the well-documented elevated stress among parents of autistic children, little is known about how these experiences of stress differ by parent gender in preschool children. Emerging research suggests that mothers and fathers may encounter distinct stressors and coping challenges due to differing caregiving roles, societal expectations, and support accessibility (Davis & Carter, 2008; Hastings et al., 2005). While studies have explored high maternal stress due to higher levels of emotional and caregiving burden (Plant & Sanders, 2007), fathers’ experiences of stress are often underreported, partly due to a relative scarcity of research focusing specifically on paternal perspectives in families of autistic children (Johnson & Simpson, 2013). Thus, these gender-specific stress patterns are still insufficiently understood, hindering the development of targeted interventions tailored to the unique needs of both mothers and fathers. In addition, most existing studies exploring the relationship between parental stress and autistic traits tend to be small scale, limiting the generalisability of their findings.

To address this knowledge gap, the present study aimed to use a large Australian national sample to determine the social and clinical indicators associated with stress among parents of autistic children, with a particular focus on identifying any gender-specific determinants. Understanding these differences is essential to inform targeted and holistic approaches that address the complex dynamics of stress within families raising autistic children.

## Methods

### Study Design and Participants

Study findings are reported following the Strengthening the Reporting of Observational Studies in Epidemiology reporting guidelines (Von Elm et al., 2014). This is a secondary analysis of data sample of 516 unique children collected as part of the Autism subtyping project–a prospective, multisite study aimed to determine the predictors of early intervention outcomes for preschool children receiving Early Intensive Intervention through six Autism Specific Early Learning and Care Centres across Australia. After obtaining formal written consent, all the children underwent clinical assessment to determine the differences associated with autistic features. In addition, parents completed questionnaires regarding their child’s autistic behaviours, personal experiences of caregiving, stress and quality of life, and relevant sociodemographic information. More information about the programmes, assessments, and follow-up are detailed elsewhere (Masi et al., 2021).

### Data Collection

#### Outcome Measure

The **Parental Stress Index–Fourth Edition–Short Form** (PSI-4-SF) is a 36-item self-reported questionnaire that is used to assess parental perceptions of stress related to their responsibilities as caregivers (Abidin, 2012). This questionnaire covers three subscales of parental distress, parent-child dysfunctional interaction, and perceived difficulties in child characteristics, with higher scores indicating higher level of perceived stress.

For the purpose of this study, we used both the subscales and the total stress scores as outcome variables and limited the sample to those parents who answered this questionnaire. In some cases, both parents of the same child participated, while in others only one parent completed the measure, providing a broad representation of parental perspectives from both mothers and fathers. In this sample, there was excellent internal consistency with Cronbach’s alpha of 0.93.

#### Exposure Variables

**The Quality of Life in Autism Questionnaire (QOLA)** is a two-part (Parts A and B) questionnaire for parents of autistic children to self-report their quality of life (Eapen et al., 2014). Part A measures the parent’s subjective wellbeing with scores ranging from 28 to 140 while Part B rates the parent’s perception of the impact on quality of life based on items specific to their autistic child with scores ranging from 20 to 100. Raw scores were used with higher scores in both parts indicate better quality of life. In this sample, there was excellent internal consistency with Cronbach’s alpha of 0.94 for Part A and 0.93 for Part B.

**The Autism Diagnostic Observation Schedule – Second Edition (ADOS-2)** is considered one of the gold standard tools for a diagnosis of ASD (Rutter et al., 2012). This clinician-reported assessment includes multiple modules designed to match an individual’s age and language abilities, allowing its use across a wide range of developmental stages. For this study’s sample of children aged 1–5 years, the Toddler Module and Modules 1–3 were administered based on verbal comprehension level. Raw scores were converted to calibrated severity scores to enable comparability across modules, with higher scores reflecting greater differences in autistic features. In this sample, there was good internal consistency with Cronbach’s alpha of 0.84.

**The Child Behaviour Checklist (CBCL)** is a questionnaire completed by parents of children to assess their behaviours and social interactions (Achenbach, 2001) and in this study, the 1.5 to 5 year version was used. Behaviours observed are characterised into internalising and externalising symptoms, including mood, attention, aggression, somatic symptoms, social problems, thought and rule-breaking behaviours etc., aligned to various mental health conditions as per the *Diagnostic and Statistical Manual of Mental Disorders* (DSM)-orientated symptoms. Higher scores indicate increased emotional and behavioural problems in everyday life. In this sample, there was excellent internal consistency with Cronbach’s alpha of 0.96.

**The Social Communication Questionnaire (SCQ)** is used to assess the current and developmental presentations of children with a mental age over 2 years (Rutter, 2003). Raw scores were used, with higher scores indicating increased difficulties with social communication and a higher risk of being autistic. In this sample, there was excellent internal consistency with Cronbach’s alpha of 0.92.

**The Repetitive Behaviours Scale-Revised (RBS-R)** is a parent-report questionnaire of their child’s restrictive or repetitive behaviour or interests (Lam & Aman, 2007). It is used to ascertain stereotyped behaviour, self-injurious behaviour, compulsive behaviour, routine, sameness, restricted behaviour as well as a total problem score. Raw scores were used, with higher scores indicating greater difficulties. In this sample, there was excellent internal consistency with Cronbach’s alpha of 0.94.

**The Vineland Adaptive Behaviour Scales, Second Edition (VABS-2)** is a semi-structured interview or a questionnaire format to measure a child’s range of adaptive and coping behaviours (Sparrow, 2011). It consists of five domains of communication, daily living skills, socialisation, motor skills, and maladaptive behaviours, with higher scores indicative of better adaptive behaviours. It can help ascertain the adaptive level of functioning and is useful for informing the plans for intervention and supports. In this sample, there was good internal consistency with Cronbach’s alpha of 0.84.

**The Mullen Scales of Early Learning (MSEL)** is an assessment tool that assesses cognitive function for children under 68 months (Mullen, 1995). A series of interactive activities measure sensory learning abilities in fine and gross motor skills, visual reception, and receptive and expressive language, with higher scores indicating better developmental functioning. It is often used in autistic preschool children for assessing intellectual development and is vital for the evaluation of ‘school readiness’ and the implementation of early intervention programmes. In this sample, there was excellent internal consistency with Cronbach’s alpha of 0.90.

**The key sociodemographic indicators** measured in the study consisted of both child and parental characteristics. Parental characteristics measured carers’ age (in years), culturally and linguistically diverse (CALD) status based on ‘language spoken at home’ to be non-English and/or the ‘country of birth’ was a non-English-speaking country (yes or no), disability status (yes or no), education level (primary/secondary level, tertiary/graduate level), occupation type (professional/paraprofessional, other) and annual income (up to $40 K, $40 K to 100 K, above $100 K). Child-related factors included age (in years), gender (male, female), first born status (yes or no), presence of co-occurring conditions (yes or no), having an autistic sibling (yes or no).

### Data Analysis

Eligibility criteria for the analyses included data from 465 mothers and 216 fathers who completed the PSI-4-SF questionnaire. Sample characteristics were analysed using descriptive statistics and presented as mean with standard deviations for continuous measures and as frequency counts with percentages for categorical measures. Pearson’s correlation analysis was conducted to determine any significant correlation between each of the clinical indicators and sociodemographic variables.

Primary analysis included multilevel multivariable regression analyses to examine the association between sociodemographic factors, parental quality of life, child’s autistic traits, and parental stress (domains and total stress scores). Analyses were stratified by parental gender, with separate models estimated for mothers and fathers. Sociodemographic factors were included as covariates in all models. To address non-independence of multiple parents reporting on the same child, models included random intercepts at the child level, ensuring unbiased estimates and valid standard errors. Findings of the regression models were reported as standardised beta coefficient (β), confidence interval (CI) and *p*-value (*p*). All statistical analyses were performed using Statistical Package for Social Sciences (SPSS) v28 (SPSS for MacOS, SPSS Inc., Chicago, IL, USA) and the R language v3.6.1 within the RStudio IDE.

## Results

### Sociodemographic and Clinical Characteristics

The sociodemographic and sociocultural characteristics of parents and children are presented in Tables 1 and 2. Of note, about one in two parents were from a CALD background. In terms of parental quality of life, both parents reported similar quality of life with no significant difference. However, mothers reported higher levels of mean total stress scores compared to fathers (mean scores of 99.09 ± 21.81 vs 96.69 ± 21.86), particularly significant in the domain of parental distress (33.03 ± 9.75 vs 31.15 ± 9.69, *p* = 0.019).

**Table 1.** Parent Sociodemographic Characteristics and Self-Reported Assessments by Gender.

Parent-level characteristics	Mothers (*N* = 465) *N* (%) or mean (*SD*)	Fathers (*N* = 216) *N* (%) or mean (*SD*)	*p*-value
**Sociodemographic characteristics**			
Age, mean (*SD*)	34.9 (5.42)	37.55 (6.54)	<0.001
CALD status			0.030
*Non-CALD*	225 (53.7)	83 (44.1)	
*CALD*	194 (46.3)	105 (55.9)	
*Missing*	78 (11.4)		
Education level			0.116
*Primary/secondary level*	106 (23.8)	57 (29.7)	
*Tertiary/graduate level*	340 (76.2)	135 (70.3)	
*Missing*	47 (6.9)		
Occupation			<0.001
*Professional/Paraprofessional*	138 (32.5)	88 (48.1)	
*Other*	287 (67.5)	95 (51.9)	
*Missing*	77 (11.2)		
Carer’s disability status			0.302
*No*	287 (84.9)	137 (88.4)	
*Yes*	51 (15.1)	18 (11.6)	
*Missing*	192 (28.0)		
*Annual income*			0.673
*Up to AU$40* *K*	83 (25.2)	39 (23.5)	
*AU$40* *K to 100* *K*	129 (39.2)	72 (43.4)	
*Above AU$100* *K*	117 (35.6)	55 (33.1)	
*Missing*	190 (27.7)		
**Self-reported assessments**			
Quality of Life in Autism (QoLA)			
*Part A*	96.14 (19.66)	96.72 (20.92)	0.730
*Part B*	69.96 (20.99)	67.48 (21.66)	0.162
Parental Stress Index (PSI)			
*Parental distress*	33.03 (9.75)	31.15 (9.69)	0.019
*Difficult child*	31.64 (7.74)	31.70 (8.09)	0.925
*Parent–Child dysfunctional interaction*	34.41 (8.49)	33.83 (8.03)	0.403
*Total stress*	99.09 (21.81)	96.69 (21.86)	0.182

**Table 2.** Child Sociodemographic and Clinical Characteristics.

Child-level characteristics	*N*	% or mean (*SD*)
**Sociodemographic characteristics**		
Child’s age, mean (*SD*)	516	3.4 (0.9)
Child’s gender		
*Male*	411	79.7
*Female*	105	20.3
First born child		
*No*	269	52.1
*Yes*	238	46.1
*Missing*	9	1.7
Other medical conditions		
*No*	235	45.5
*Yes*	281	54.5
Sibling’s ASD diagnosis		
*No*	227	44.0
*Yes*	119	23.1
*Missing/Not applicable*	170	32.9
**Clinical characteristics**		
Autism Diagnostic Observation Schedule – Second Edition (ADOS-2)		
*Calibrated severity scores*	427	7.2 (1.8)
Child Behavioural Checklist (CBCL)		
*Internalising problem score*	516	59.4 (10.6)
*Externalising problem score*	516	63.4 (8.9)
Social Communication Questionnaire (SCQ) total score	481	19.0 (6.0)
Repetitive Behaviours Scale – Revised (RBS-R) total score	123	48.5 (27.1)
Vineland Adaptive Behaviour Scale (VABS-2)^a^		
*Communication*	419	66.2 (16.8)
*Daily living skills*	516	56.2 (31.3)
*Socialisation*	516	55.3 (29.2)
*Motor skills*	516	61.2 (33.3)
Mullen Scales of Early Learning (MSEL) verbal developmental quotient (VDQ)	516	45.7 (26.8)
Mullen Scales of Early Learning (MSEL) non-verbal developmental quotient (NVDQ)	516	59.0 (22.9)

a Domain standard scores.

In relation to children’s characteristics (Table 2), most of the children were male (79.7%) and over half of the children (54.5%) had been found to have one or more co-occurring conditions, and almost a quarter (23.5%) reported having an autistic sibling. In terms of clinical measures in Table 2, children displayed moderate levels of differences in autistic features with mean calibrated severity scores of 7.2 ± 1.8.

### Factors Associated with Maternal Stress

Findings of the multilevel linear regression models showing risk factors of maternal stress are presented in Table 3. In terms of sociodemographic predictors, CALD status and higher levels of education were found to be significant protective factors against maternal stress. Compared to mothers from non-CALD backgrounds, those from CALD backgrounds reported significantly lower total stress scores (β = −7.51; 95% CI: −14.27, −0.74; *p* = 0.029) as well as in the ‘difficult child’ subscale scores (β = −3.53; 95% CI: 06.20, −0.86; *p* = 0.009). In addition, maternal education was a key factor, where mothers with lower levels of education (primary/secondary education) had higher levels of total stress (β = 11.21; 95% CI: 3.12, 19.3; *p* = 0.006) compared to those with higher levels of education. This pattern was similarly reflected in the ‘parental distress’ (β = 4.98; 95% CI: 1.47, 8.50; *p* = 0.005) and ‘difficult child’ (β = 3.68; 95% CI: 0.48, 6.87; *p* = 0.024) subscales.

**Table 3.** Multilevel Linear Regression Models Showing Risk Factors Associated with Maternal Stress.

Variables	Parental distress β (95% CI)	*p*-value	Difficult child β (95% CI)	*p*-value	Parent–Child dysfunctional Interaction β (95% CI)	*p*-value	Total stress β (95% CI)	*p*-value
**Sociodemographic covariates**								
Child’s age	−1.56 (−3.30, 0.18)	0.079	0.36 (−1.22, 1.95)	0.651	−0.20 (−1.62, 1.23)	0.787	−1.39 (−5.40, 2.62)	0.494
Child’s gender								
*Male*	Reference		Reference		Reference		Reference	
*Female*	−2.32 (−5.77, 1.12)	0.184	−0.60 (−3.73, 2.53)	0.705	0.92 (−1.90, 3.74)	0.521	−2.00 (−9.93, 5.92)	0.618
First born child								
*No*	Reference		Reference		Reference		Reference	
*Yes*	−0.02 (−1.39, 1.35)	0.979	−0.35 (−1.59, 0.90)	0.580	0.07 (−1.06, 1.19)	0.907	−0.30 (−3.45, 2.85)	0.850
Other medical conditions								
*No*	Reference		Reference		Reference		Reference	
*Yes*	0.05 (−3.21, 3.32)	0.974	0.70 (−2.27, 3.67)	0.641	0.60 (−2.08, 3.28)	0.660	1.35 (−6.16, 8.87)	0.723
Sibling’s ASD diagnosis								
*No*	Reference		Reference		Reference		Reference	
*Yes*	2.79 (−0.40, 5.97)	0.085	1.13 (−1.77, 4.03)	0.442	2.53 (−0.08, 5.15)	0.057	6.45 (−0.8, 13.79)	0.084
Parental age	0.05 (−0.23, 0.34)	0.717	−0.18 (−0.44, 0.08)	0.174	−0.05 (−0.28, 0.18)	0.684	−0.17 (−0.83, 0.48)	0.598
CALD status								
*Non-CALD*	Reference		Reference		Reference		Reference	
*CALD*	−2.10 (−5.04, 0.83)	0.159	−3.53 (−6.20, −0.86)	0.009	−1.87 (4.28, 0.54)	0.126	−7.51 (−14.27, −0.74)	0.029
Parental education level								
*Tertiary/graduate level*	Reference		Reference		Reference		Reference	
*Primary/secondary level*	4.98 (1.47, 8.50)	0.005	3.68 (0.48, 6.87)	0.024	2.55 (−0.33, 5.43)	0.082	11.21 (3.12, 19.3)	0.006
Carer’s disability status								
*No*	Reference		Reference		Reference		Reference	
*Yes*	−0.91 (−4.71, 2.90)	0.638	−2.34 (−5.80, 1.13)	0.184	−0.14 (−3.26, 2.98)	0.929	−3.38 (−12.14, 5.38)	0.447
**Maternal quality of life**								
*Part A*	−0.22 (−0.30, −0.15)	<0.001	−0.16 (−0.22, −0.09)	<0.001	−0.12 (−0.18, −0.06)	<0.001	−0.50 (−0.66, −0.33)	<0.001
*Part B*	−0.11 (−0.17, −0.05)	<0.001	−0.13 (−0.19, −0.08)	<0.001	−0.09 (−0.14, −0.04)	<0.001	−0.33 (−0.47, −0.20)	<0.001
**Child behavioural and cognitive functioning**								
Autism Diagnostic Observation Schedule – Second Edition (ADOS-2)								
*Calibrated severity scores*	0.48 (−0.42, 1.37)	0.293	0.52 (−0.29. 1.33)	0.208	0.81 (0.11, 1.51)	0.023	1.80 (−0.22, 3.83)	0.079
Child Behavioural Checklist (CBCL)								
*Internalising problem score*	0.34 (0.16, 0.52)	<0.001	0.41 (0.26, 0.57)	<0.001	0.38 (0.24, 0.52)	<0.001	1.13 (0.73, 1.52)	<0.001
*Externalising problem score*	0.24 (0.09, 0.40)	0.001	0.37 (0.24, 0.50)	<0.001	0.23 (0.10, 0.35)	<0.001	0.84 (0.50, 1.18)	<0.001
Social Communication Questionnaire (SCQ) score	0.34 (0.08, 0.59)	0.009	0.53 (0.31, 0.75)	<0.001	0.54 (0.35, 0.73)	<0.001	1.41 (0.86, 1.96)	<0.001
Repetitive Behaviours Scale – Revised (RBS-R) score	0.16 (0.05, 0.26)	0.006	0.16 (0.06, 0.25)	0.001	0.11 (0.04, 0.18)	0.004	0.42 (0.17, 0.67)	0.001
Vineland Adaptive Behaviour Scale (VABS-2)								
Communication	0.01 (−0.08, 0.10)	0.769	−0.04 (−0.12, 0.04)	0.353	−0.08 (−0.16, −0.01)	0.023	−0.11 (−0.32, 0.10)	0.295
Daily living skills	−0.01 (−0.07, 0.05)	0.789	0.03 (−0.03, 0.09)	0.288	−0.05 (−0.10, −0.00)	0.050	−0.03 (−0.17, 0.11)	0.696
Socialisation	−0.01 (−0.08, 0.05)	0.719	0.04 (−0.02, 0.10)	0.159	−0.03 (−0.09, 0.02)	0.204	−0.00 (−0.15, −0.15)	0.958
Motor living skills	−0.01 (−0.07, 0.05)	0.739	0.04 (−0.01, 0.09)	0.112	−0.04 (−0.08, 0.01)	0.119	−0.01 (−0.14, 0.13)	0.942
Mullen Scales of Early Learning (MSEL) verbal developmental quotient (VDQ)	0.04 (−0.02, 0.10)	0.151	−0.00 (−0.05, 0.05)	0.868	−0.02 (−0.07, 0.02)	0.289	0.01 (−0.12, 0.14)	0.857
Mullen Scales of Early Learning (MSEL) non-verbal developmental quotient (NVDQ)	0.05 (−0.02, 0.12)	0.160	−0.01 (−0.08, 0.05)	0.720	−0.03 (−0.09, 0.03)	0.361	0.01 (−0.15, 0.17)	0.887

Maternal quality of life and child behavioural and cognitive functioning are adjusted for sociodemographic covariates.

A strong inverse relationship was observed between quality of life and stress where higher quality of life (Part A) (β = −0.50; 95% CI: −0.66, −0.33; *p* < 0.001) along with lower perceived functional impact of ASD-specific difficulties (Part B) (β = −0.33; 95% CI: −0.47, −0.20; *p* < 0.001) were significantly associated with lower total stress scores in mothers. Similarly, this pattern was observed in all individual subscales of parental distress, difficult child, and parent–child dysfunctional interaction scores, after adjusting for sociodemographic covariates.

Greater differences in autistic features were significantly associated with increased parent–child dysfunctional interactions (β = 0.81; 95% CI: 0.11, 1.51; *p* < 0.001). In addition, both internalising (β = 1.13; 95% CI: 0.73, 1.52; *p* < 0.001) and externalising behaviours (β = 0.84; 95% CI: 0.50, 1.18; *p* < 0.001) were also predictors of total maternal stress, as well as all other domains of the PSI. Similarly, poorer social communication scores (β = 1.41; 95% CI: 0.86, 1.96; *p* < 0.001) and greater repetitive behaviours (β = 0.42; 95% CI: 0.17, 0.67; *p* = 0.001) were linked to higher stress in all the subscales and total scores. In contrast, better adaptive functioning, particularly in communication (β = −0.08; 95% CI: −0.16, −0.01; *p* = 0.023) and daily living skills (β = −0.05; 95% CI: −0.10, −0.00; *p* = 0.050) were significantly linked to lower stress in the ‘Parent–Child dysfunctional interaction’ subscale.

### Factors Associated with Paternal Stress

Findings of the multilevel multivariable linear regression models showing risk factors of paternal stress is detailed in Table 4. Consistent with maternal findings, CALD status was protective, but only for the ‘parental distress’ subscale (β = −5.18; 95% CI: −9.78, −0.58; *p* = 0.027). In addition, child’s age was inversely associated with paternal stress, with older children linked to lower total stress scores (β = −7.62; 95% CI: −14.86, −0.47; *p* = 0.039) and lower ‘parent–child dysfunction’ scores (β = −3.35; 95% CI: −6.09, −0.60; *p* = 0.017).

**Table 4.** Multilevel Linear Regression Models Showing Risk Factors Associated with Paternal Stress.

Variables	Parental distress β (95% CI)	*p*-value	Difficult child β (95% CI)	*p*-value	Parent–Child dysfunctional Interaction β (95% CI)	*p*-value	Total stress β (95% CI)	*p*-value
**Sociodemographic covariates**								
Child’s age	−2.52 (−5.64, 0.61)	0.112	−1.75 (−4.31, 0.81)	0.176	−3.35 (−6.09, −0.60)	0.017	−7.62 (−14.86, −0.37)	0.039
Child’s gender								
*Male*	Reference		Reference		Reference		Reference	
*Female*	0.56 (−4.94, 6.06)	0.839	2.08 (−2.42, 6.58)	0.360	3.34 (−1.49, 8.17)	0.172	5.97 (−6.77, 18.72)	0.352
First born child								
*No*	Reference		Reference		Reference		Reference	
*Yes*	1.12 (−0.81, 3.05)	0.251	−0.49 (−2.07, 1.09)	0.537	−0.07 (−1.77, 1.63)	0.934	0.56 (−3.92, 5.04)	0.804
Other medical conditions								
*No*	Reference		Reference		Reference		Reference	
*Yes*	0.39 (−4.64, 5.42)	0.877	0.48 (−3.64, 4.59)	0.818	0.01 (−4.41, 4.42)	0.997	0.88 (−10.78, 12.53)	0.881
Sibling’s ASD diagnosis								
*No*	Reference		Reference		Reference		Reference	
*Yes*	2.64 (−2.85, 8.12)	0.340	0.10 (−4.39, 4.58)	0.966	0.69 (−4.13, 5.50)		3.42 (−9.29, 16.12)	0.593
Parental age	0.18 (−0.22, 0.59)	0.366	−0.08 (−0.41, 0.25)	0.636	−0.00 (−0.36, 0.35)	0.992	0.10 (−0.83, 1.04)	0.826
CALD status								
*Non-CALD*	Reference		Reference		Reference		Reference	
*CALD*	−5.18 (−9.78, −0.58)	0.027	−2.46 (−6.22, 1.31)	0.197	−1.97 (−6.01, 2.06)	0.333	−9.61 (−20.26, 1.05)	0.076
Parental education level								
*Tertiary/graduate level*	Reference		Reference		Reference		Reference	
*Primary/secondary level*	3.26 (−1.84, 8.35)	0.205	0.93 (−3.24, 5.10)	0.659	−0.21 (−4.69, 4.26)	0.924	3.97 (−7.83, 15.78)	0.504
Carer’s disability status								
*No*	Reference		Reference		Reference		Reference	
*Yes*	−1.20 (−7.59, 5.19)	0.709	0.14 (−5.10, 5.37)	0.959	−1.52 (−7.13, 4.09)	0.590	−2.59 (−17.40, 12.23)	0.728
**Paternal quality of life**								
*Part A*	−0.25 (−0.35, −0.16)	<0.001	−0.08 (−0.18, 0.01)	0.070	−0.13 (−0.22, −0.03)	0.009	−0.46 (−0.70, −0.22)	<0.001
*Part B*	−0.12 (−0.22, −0.01)	0.026	−0.08 (−0.16, 0.01)	0.068	−0.10 (−0.19, −0.01)	0.028	−0.29 (−0.53, −0.06)	0.014
**Child behavioural and cognitive functioning**								
Autism Diagnostic Observation Schedule – Second Edition (ADOS-2)								
*Calibrated severity scores*	−0.36 (−1.68, 0.96)	0.585	−0.77 (−1.80, 0.27)	0.142	0.15 (−1.01, 1.30)	0.798	−0.98 (−3.97, 2.00)	0.511
Child Behavioural Checklist (CBCL)								
*Internalising problem score*	0.36 (0.08, 0.65)	0.013	0.25 (0.01, 0.49)	0.040	0.46 (0.22, 0.70)	<0.001	1.07 (0.42, 1.72)	0.001
*Externalising problem score*	0.34 (0.10, 0.59)	0.005	0.33 (0.14, 0.52)	0.001	0.34 (0.13, 0.55)	0.001	1.01 (0.47, 1.55)	<0.001
Social Communication Questionnaire (SCQ) score	0.17 (−0.18, 0.51)	0.345	−0.04 (−0.32, 0.24)	0.767	0.33 (0.03, 0.62)	0.029	0.45 (−0.33, 1.23)	0.254
Repetitive Behaviours Scale – Revised (RBS-R) total score	0.08 (−0.15, 0.31)	0.457	0.01 (−0.20, 0.22)	0.938	0.05 (−0.13, 0.23)	0.559	0.13 (−0.37, 0.64)	0.568
Vineland Adaptive Behaviour Scale (VABS-2)								
Communication	0.04 (−0.12, 0.21)	0.615	0.01 (−0.12, 0.14)	0.903	−0.04 (−0.12, 0.05)	0.241	−0.03 (−0.41, 0.34)	0.864
Daily living skills	0.01 (−0.09, 0.12)	0.796	0.04 (−0.05, 0.13)	0.338	−0.08 (−0.22, 0.06)	0.225	−0.00 (−0.25, 0.25)	0.993
Socialisation	−0.01 (−0.11, 0.10)	0.860	0.03 (−0.05, 0.12)	0.429	−0.06 (−0.15, 0.04)	0.152	−0.04 (−0.28, 0.20)	0.737
Motor living skills	0.01 (−0.08, 0.11)	0.772	0.04 (−0.04, 0.12)	0.305	−0.07 (−0.16, 0.03)	0.402	0.02 (−0.20, 0.24)	0.865
Mullen Scales of Early Learning (MSEL) verbal developmental quotient (VDQ)	0.03 (−0.05, 0.11)	0.464	0.02 (−0.05, 0.09)	0.531	−0.01 (−0.08, 0.07)	0.873	0.05 (−0.14, 0.23)	0.634
Mullen Scales of Early Learning (MSEL) non-verbal developmental quotient (NVDQ)	0.03 (−0.08, 0.14)	0.574	0.04 (−0.05, 0.13)	0.393	−0.01 (−0.11, 0.08)	0.806	0.06 (−0.19, 0.31)	0.652

Paternal quality of life and child behavioural and cognitive functioning are adjusted for sociodemographic covariates.

Consistent inverse relationship was observed where higher quality of life and greater adaptability in fathers was significantly associated with lower total stress (β = −0.46; 95% CI: −0.70, −0.22; *p* < 0.001) and lower scores in the ‘parental distress’ and ‘parent–child dysfunction’ subscales. However, unlike mothers, quality of life was not significantly associated with the ‘difficult child’ subscale in fathers, but was significant in other subscales.

Only a limited set of child’s autistic traits were linked to paternal stress. Increased internalising (β = 1.07; 95% CI: 0.42, 1.72; *p* = 0.001) and externalising child behaviours (β = 1.01; 95% CI: 0.47, 1.55; *p* < 0.001) were associated with higher stress within all domains. Interestingly, poorer social communication was not associated with total paternal stress but only with parent–child dysfunction (β = 0.33; 95% CI: 0.14, 0.52; *p* = 0.001). There were no other significant associations between child’s repetitive behaviours or adaptive functioning and paternal stress.

## Discussion

This study examined the complex relationship between sociodemographic factors, child’s autistic traits, and stress in parents of autistic preschool children with a gender-specific lens. A key strength of this study is the inclusion and analysis of both maternal and paternal data, which remains relatively rare in the literature. Fathers have been traditionally underrepresented in research, often due to lower participation rates or study designs that assume mothers as the primary caregiver. Our study provides valuable insights into gender-specific caregiving experiences by separately modelling factors associated with maternal and paternal stress. This has implications for developing targeted interventions and supports that respect the differential impact of ASD-related stressors on mothers and fathers.

Importantly, these findings are supported by the strong reliability of the parental stress measure (PSI-4-SF), which demonstrated excellent internal consistency in this sample, which includes approximately 50% of parents from CALD backgrounds. While this supports the measure’s reliability for parents of autistic preschool children, cultural factors may still influence how parents perceive and report stress, highlighting the need for cautious interpretation and further research on the measure’s performance across diverse populations (Gao & Lee, 2021; González-López et al., 2024).

CALD status emerged as a protective factor against multiple subscales of stress among both mothers and fathers. The protective effect was broader for mothers, with significant effect in both total stress and ‘difficult child’ subscales scores, and only for the parental distress subscale in fathers. This finding may appear counterintuitive, as existing literature often reports CALD status as a potential barrier to service access and mental health support (Said et al., 2021; Wohler & Dantas, 2017). From a cultural and resilience perspective, these results are consistent with collectivist values often found in many CALD communities, where shared caregiving, strong family bonds and extended social supports, and a greater acceptance of differences can offer important protective benefits (Renzaho et al., 2011; Smith et al., 2021). These family characteristics may not only alleviate the burden of caregiving but also reduce internalised stigma and caregiver guilt. In addition, coping strategies influenced by religious or spiritual beliefs, culturally grounded expectations of child development, and communal narratives of resilience may moderate the psychological impact associated with caregiving (Stahmer et al., 2019). These insights underscore the importance of designing interventions that leverage culturally embedded strengths, rather than positioning CALD status solely as a risk factor.

Higher level of maternal education was protective against stress across multiple domains of the PSI, possibly due to greater access to resources, higher health literacy, and improved problem-solving skills. Mothers with higher levels of education may navigate caregiving challenges more effectively, advocate for their child’s needs with confidence, and experience less financial strain, all of which help reduce parenting stress (Parkes et al., 2015). On the other hand, the association between education level and stress was not significant for fathers, possibly reflecting differences in caregiving roles, interaction patterns, or the relative weight of daily caregiving demands in their stress experience. For fathers, child’s age was a protective factor, with older children associated with lower stress levels, particularly in the parent–child dysfunction domain. As children develop greater independence and adaptive skills, fathers may experience reduced daily caregiving demands, which can lower stress levels (Piotrowski et al., 2023).

Parental quality of life was another consistent predictor of lower stress for both mothers and fathers. Higher wellbeing (QoLA Part A) and lower impact of autism-specific challenges (QoLA Part B) were strongly associated with lower total stress and reduced strain across most PSI domains (John et al., 2025). The association was particularly pronounced for mothers, extending to all subscales, while for fathers, the relationship did not extend to the ‘difficult child’ domain. This suggests that behavioural challenges may remain a source of stress for fathers regardless of their overall wellbeing (Ping et al., 2023).

The impact of child’s behavioural, emotional, and cognitive challenges was notably broader for mothers compared to fathers. For mothers, higher stress was linked not only to behavioural difficulties but also to greater differences in autistic features, poorer social communication, increased repetitive behaviours, and lower adaptive functioning in communication and daily living skills. This heightened sensitivity may reflect mothers’ predominant role as primary caregivers, leading to more sustained exposure to daily caregiving challenges (Soltanifar et al., 2015). It may also reflect societal expectations that mothers take primary responsibility for fostering their child’s development, thereby impacting their stress levels (Papadopoulos, 2021). On the other hand, fathers’ stress was linked only to child’s internalising and externalising behaviours and not with other traits such as repetitive behaviours or adaptive functioning. These differences may suggest that fathers are less directly affected by certain aspects of autistic features, potentially due to variations in caregiving responsibilities, interaction patterns, or developmental expectations (Ozturk et al., 2014).

### Implications for Policy and Practice

Findings from this study highlight the need for more targeted, family-centred supports in early intervention and support services. Screening tools and interventions should account for the differential needs of both fathers and mothers, considering the unique sociodemographic and clinical risk factors, to more effectively reduce stress and promote wellbeing. Future interventions could include paternal-focused components addressing behavioural management, supporting parent–child interactions, and fostering developmental gains, while maternal-focused interventions may target broader developmental and behavioural challenges, enhance coping strategies, and develop problem-solving skills. Policy makers should also prioritise inclusive models of care that engage CALD families and leverage their cultural strengths using strength-based approaches (Sege & Browne, 2017). Parental mental health should be routinely monitored alongside child outcomes in early intervention settings to ensure a holistic family-centred care.

### Strengths, Limitations and Directions for Future Research

Strengths of this study included the use of a large, national sample of parents from diverse sociodemographic backgrounds across multiple Australian sites. The inclusion of both mothers and fathers allowed for a gendered analysis of parental stress, an area often underrepresented in autism research. In addition, the use of multilevel modelling provided a more nuanced understanding of the determinants of parental stress. However, several limitations should be acknowledged. First, the cross-sectional design limits the ability to draw causal inferences between child’s autistic traits and behaviours, sociodemographic factors, and parental stress. Second, the study sample may be subject to selection bias as it comprised families engaged in formal early intervention services, and potentially limiting the generalisability of findings to the broader population of parents of autistic children. Third, several measures relied on self-report questionnaires, which are susceptible to social desirability and recall bias. This limitation may be particularly relevant for fathers, where societal norms around stoicism could lead to underreporting of stress, or conversely, over-reporting by mothers due to heightened awareness and anxiety about their child’s behaviours.

Future research should employ longitudinal designs to explore how parental stress and its predictors evolve over time, particularly in response to early intervention. Tracking changes in stress as children transition through developmental stages could clarify causality and identify critical intervention windows. In addition, qualitative research involving in-depth interviews with mothers and fathers would provide richer insights into caregiving experiences, perceptions of support, and cultural influences. Given the protective role of CALD status found in this study, future research should investigate the specific mechanisms of resilience in CALD families such as coping mechanisms, family structures, and cultural narratives that may underlie these patterns, using detailed methods, including in-depth interviews or focus groups.

## Conclusion

This study provides new insights into the gender and contextual determinants of stress among mothers and fathers of autistic children in Australia. While internalising and externalising behaviours were consistently linked to stress among both mothers and fathers, mothers experienced additional challenges related to communication, repetitive behaviours, and adaptive functioning challenges in their children. Sociodemographic factors such as CALD background and higher levels of education appeared protective, particularly for mothers. These findings underscore the need to leverage cultural resilience and for early, personalised, and culturally sensitive interventions and supports that not only target child outcomes but also prioritise caregiver wellbeing. Supporting families more effectively will ultimately benefit autistic children as they navigate developmental and social milestones while also ensuring parental wellbeing and quality of life.
